# Immune-mediated skin diseases and erectile dysfunction: mechanisms and multidisciplinary management

**DOI:** 10.3389/fimmu.2026.1704717

**Published:** 2026-02-02

**Authors:** Chengsen Lv, Hongliang Cao, Dingliang Zhao, Yongjin Yang, Zhen Ma, Yinuo Zhang, Xingyu Wu, Mo Chen, Jialin Gao

**Affiliations:** 1 Department of Urology, The First Hospital of Jilin University, Changchun, China; 2 Department of Urology, The Second Hospital of Jilin University, Changchun, China; 3 Department of Breast Surgery, General Surgery Center, The First Hospital of Jilin University, Changchun, China

**Keywords:** erectile dysfunction, immune-mediated skin diseases, multidisciplinary management, screening, treatment

## Abstract

The global prevalence of erectile dysfunction (ED) continues to rise, which has become an important issue affecting the physical and mental health of men. Existing evidence suggests that ED is closely related to various immune-mediated skin diseases. This review elaborates on the epidemiological characteristics, potential mechanisms, and clinical management strategies of ED associated with immune-mediated skin diseases. It discusses the future research directions and challenges in this field. Epidemiological studies consistently show that the prevalence of ED in such patients is higher than that in the general population, indicating that immune-mediated skin diseases may significantly increase the risk of ED. Its pathogenesis is complex and diverse, involving the interaction of multiple pathways. In terms of clinical management, a multidisciplinary collaborative model is advocated, with the active control of the inflammatory activity of the primary skin disease as the cornerstone of treatment. The treatment of ED should be individualized, combined with psychological intervention and lifestyle optimization, to comprehensively improve the treatment effect. Future research should further explore its molecular mechanisms and conduct large-sample, prospective clinical trials to optimize treatment strategies and ultimately enhance patients’ sexual health and overall quality of life (QoL).

## 1 Introduction

Erectile Dysfunction (ED) refers to a man’s persistent or recurrent inability to achieve or maintain a penile erection of sufficient firmness to complete satisfactory sexual intercourse (1). It is a common male sexual health disorder. In recent years, the prevalence of this disease has increased globally. Its causes are relatively complex, often involving the combined effects of multiple mechanisms such as organic factors, psychological factors, lifestyle, and the effects of medications (2). ED not only seriously affects the quality of sexual life of patients and their partners but may also damage the patients’ self-confidence, mental health, and the stability of interpersonal relationships. Due to the personal nature of the issue, many patients avoid seeking medical help out of shame, thereby explaining why ED is commonly overlooked or inadequately treated despite its prevalence (3). In addition, ED is no longer just an issue related to sexual activity. Growing evidence shows that ED is not only an important early manifestation of diseases such as cardiovascular diseases and diabetes but is also associated with certain skin-related immune diseases (4, 5). Therefore, early diagnosis and active intervention for ED are of great clinical significance.

The skin is the largest organ of the human body. It not only forms the first physical barrier for the body to resist the external environment but also serves as a highly active immune interface, undertaking essential functions such as immune surveillance, defense against pathogens, and maintenance of internal environment homeostasis (6). The immune system plays a central role in maintaining the health of the body and can recognize and eliminate foreign pathogens. However, when the regulatory mechanism of the immune system malfunctions and mistakenly attacks the body’s own healthy skin cells or tissues, it is implicated in the pathogenesis of a variety of immune-mediated skin diseases (7). Although the clinical manifestations of such diseases are concentrated on the skin, they essentially reflect an imbalance in immune homeostasis and may be accompanied by the involvement of other organs. The skin immune system has a complex structure and precise functions, and its dysfunction is the pathological basis for many diseases with heterogeneous clinical manifestations. These diseases usually involve multiple pathophysiological pathways, including chronic inflammation, autoimmune responses, and allergic reactions. Most of them follow a chronic and recurrent course and are easily induced or aggravated by factors such as infection, stress, sunlight, or drugs (8, 9). At present, the vast majority of immune-mediated skin diseases are still difficult to cure completely, and treatment relies on long-term and comprehensive management strategies (10). It is worth noting that in recent years, excellent reviews have respectively elaborated on the epidemiological associations between specific immune-mediated skin diseases (such as psoriasis and atopic dermatitis) and ED (11, 12). However, there is still a lack of review literature that takes such diseases as an overall category, systematically compares the common mechanisms and disease-specific differences in their associations with ED, and constructs a cross-disease comprehensive management framework on this basis. This paper aims to make up for this deficiency, provide new ideas for the prevention and treatment of ED, and offer a reference for the diagnosis and treatment practice of related diseases.

## 2 Literature search and selection methodology

To comprehensively summarize the association between immune-mediated skin diseases and ED, we searched the PubMed database for relevant literature published from its inception to August 2025. The search scope covered various immune-mediated skin diseases. A comprehensive search was conducted for each disease by combining “ED” with its corresponding Medical Subject Headings (MeSH) terms and free terms. The inclusion criteria for the literature were observational studies, randomized controlled trials, systematic reviews, and meta-analyses published in English. After careful screening, the literature closely related to the topic was finally selected for evidence integration and mechanism exploration.

This review focuses on psoriasis, hidradenitis suppurativa (HS), scleroderma, systemic lupus erythematosus (SLE), atopic dermatitis (AD), Behçet’s disease (BD), and lichen sclerosus (LS), mainly based on the following considerations. First, there is substantial epidemiological and mechanistic evidence in the existing literature on the association between these diseases and ED, providing a sufficient basis for analysis. Second, in male patients, these diseases not only show a relatively high comorbidity rate of ED, but ED is often related to the severity of the diseases, highlighting the urgency of clinical recognition and management. Finally, these diseases can represent different pathophysiological patterns by which immune-mediated skin diseases affect ED, which helps to construct an analysis framework with relatively comprehensive mechanism coverage. The selection in this article aims to provide a discussion based on current evidence and centered on core clinical issues, rather than an exhaustive list of all immune-mediated skin diseases.

## 3 Epidemiological evidence of the association between immune-mediated skin diseases and ED

Data from recent observational studies indicate that there are differences in sexual function, reproductive hormones, and sperm quality between patients with immune-mediated skin diseases and healthy men. This not only suggests that immune-mediated skin diseases may play an essential role in the risk of occurrence, progression, and poor clinical outcomes of ED but also highlights the urgency of early identification and systematic management of this group of people (**Table 1**).

**Table 1:** Main characteristics of epidemiological studies on immune-mediated skin diseases and ED.

Authors, year	Disease	Number of cases, mean age (years)	Number of controls, mean age (years)	Outcome measures	Main outcomes
Li et al, 2025 (14)	LS	12, 56.8 ± 6.8	NA	Successful sexual intercourse episodes	(1) Transurethral annular inlay oral mucosa urethroplasty can improve sexual function and the appearance of the glans penis (2). Seven patients (58.3%) successfully resumed sexual activity within one year.
Varkal et al, 2024 (15)	BD	69, 39.55 ± 11.7	74, 36.9 ± 6.84	IIEF, SF-36	(1) 74% (49 cases) had sexual dysfunction (2). 17.1% of male patients with BD had low testosterone levels.
Nowowiejska et al, 2024 (16)	Psoriasis	80, 34.29 ± 1.87	75, 29.87 ± 2.05	IIEF	(1) Disease duration is negatively correlated with erectile function, confidence in maintaining erections, and frequency of sexual intercourse.
Krajewski et al, 2024 (17)	HS	119, 40.6 ± 10.3	NA	IIEF, HADS	(1) Severe ED and concomitant depression reduced the total IIEF score of patients (2). 63.8% (30 cases) of men had ED, among whom those with severe ED accounted for 70% (21 cases).
Ismail et al, 2024 (18)	Psoriasis	30, 44.03 ± 3.59	30, 44.80 ± 3.17	IIEF, BMI, MeTS components	(1) Losing weight through a healthy diet and exercise can improve the erectile function of patients with ED.
Avci İ et al, 2024 (19)	BD	50, 38.28 ± 9.21	50, 39.34 ± 7.23	IIEF, BDI	(1) 48% (n=10) of male patients had ED (2). Scores for erectile function, orgasmic function, sexual desire, and sexual satisfaction correlated with BDI scores.
Ureña-Paniego et al, 2023 (20)	AD	32, 30.53 ± 14.48	NA	IIEF, PSQI, DLQI	(1) 66.67% of male participants were assessed as having ED (2). Dupilumab treatment not only improved patients’ sexual function but was also associated with enhanced sleep and QoL.
Kędra et al, 2022 (21)	Psoriasis	109, 48.0 ± 13.4	NA	IIEF, DLQI	(1) Over 90% of respondents reported decreased attraction (2). More than 50% indicated they sometimes avoid sexual contact (3). Nearly 40% of participants frequently or persistently felt ashamed in front of their sexual partners.
Krittian et al, 2021 (22)	SSc	64, 52.3 ± 10.75	123, 52.9 ± 11.01	IIEF, BDI	(1) 22.0% of patients experienced decreased self-confidence (2). 41.5% of SSc patients reported decreased libido, with a prevalence of severe ED reaching 47.5% (3). Patients with lower IIEF scores had higher BDI scores (4). ED was associated with more severe SSc organ involvement.
Alariny et al, 2019 (23)	Psoriasis	220, 34.33 ± 8.23	220, 34.20 ± 8.21	IIEF, DLQI	(1) Sexual intercourse frequency decreased after illness onset, negatively impacting sexual life.
Eltaweel et al, 2018 (24)	Psoriasis	50, 47.64 ± 13.39	30, 45.93 ± 16.36	IIEF	(1) Serum total testosterone levels decrease, while estradiol levels increase (2). IIEF scores show a declining trend with increasing age.
Alavi et al, 2018 (25)	HS	50, 35.98 ± 13.62	50, 39.80 ± 11.80	IIEF, DLQI, SQoLM	(1) Sexual dysfunction is the main factor contributing to the decline in the QoL of male HS patients.

ED, erectile dysfunction; LS, lichen sclerosus; BD, behçet’s disease; HS, hidradenitis suppurativa; AD, atopic dermatitis; SSc, systemic sclerosis; NA, not available; IIEF, International Index of Erectile Function; SF-36, 36 Health Survey Questionnaire; HADS, Hospital Anxiety and Depression Scale; BMI, body mass index; MeTS, metabolic syndrome; BDI, Beck Depression Inventory; PSQI, Pittsburgh Sleep Quality Index; DLQI, Dermatology Life Quality Index; SQoLM, Sexual Quality of Life Questionnaire for Use in Men; QoL, quality of life.

### 3.1 Psoriasis

Psoriasis is a common, chronic, and recurrent autoimmune skin disease (13). There are multiple clinical subtypes of psoriasis, among which psoriasis vulgaris is the most common. Its typical skin lesions are well-demarcated red plaques covered with silvery-white scales. It often occurs on the scalp, extensor sides of the extremities, lumbosacral region, and back (26). A study involving 80 patients with psoriasis and 75 controls without skin diseases showed that, compared with the control group, psoriasis patients had significantly lower overall satisfaction with sexual life and less confidence in maintaining an erection (16). Another cross-sectional study analyzing 100 patients found that the location of psoriatic skin lesions affects sexual function problems, especially when the genital area, face, and hands are involved (21). A meta-analysis covering eight studies with 39,490 patients with psoriasis indicated an increased risk of ED in patients with psoriasis: odds ratio (OR)=1.62, 95% confidence interval (CI)=1.37-1.91, P < 0.001 (27). In conclusion, multiple observational studies suggest that there is an association between psoriasis and an increased risk of ED. Although residual confounding cannot be excluded entirely, these findings suggest that clinicians should pay attention to the sexual function status of psoriasis patients, especially those with skin lesions in sensitive areas such as the genitals.

### 3.2 Hidradenitis suppurativa

HS is essentially an inflammatory process triggered by the blockage and rupture of hair follicle units. This disease tends to occur in areas rich in apocrine sweat glands, such as the armpits, groin, and perianal regions (25, 28). A cross-sectional study involving 199 HS patients found that more than 60% of men had clinically significant sexual dysfunction (17). The results of multiple meta-analyses consistently show that male HS patients have more severe sexual function impairment, suggesting a correlation between HS and ED (29–31). Lotan et al.’s study on 28 male HS patients and 44 healthy controls showed that up to 93% of HS patients reported having ED, further indicating that young male HS patients may face both abnormal spermatogenesis and impaired sexual function (32). In addition, there is evidence that the severity of ED may be related to the activity and extent of HS lesions (33).

### 3.3 Connective tissue diseases

Connective tissue diseases are not a single disease, but rather a general term for a group of diseases with unclear etiologies, primarily characterized by the involvement of connective tissues in multiple organs and systems. Common ones include scleroderma, SLE, Sjögren’s disease, etc. (22, 34). These diseases typically share common characteristics with autoimmune diseases.

Scleroderma is mainly characterized by local or extensive hardening and fibrosis of the skin, as well as progressive sclerosis of internal organs. According to the scope of involvement, scleroderma can be divided into two types: localized scleroderma and systemic scleroderma (35). The lesions of localized scleroderma are mainly confined to the skin. Systemic scleroderma, also known as systemic sclerosis (SSc), not only affects the skin but can also simultaneously impact multiple organ systems. Its clinical manifestations are more extensive, and the condition is usually more severe and complex (36). A cross-sectional study of 60 adult male Thai patients with SSc showed that 53 of them met the diagnostic criteria for ED. Compared with non-ED patients, ED patients had worse orgasm, libido, and sexual intercourse satisfaction, and more severe skin sclerosis. The study indicated that ED is a common problem in male SSc patients, and the severity of SSc may increase the risk of ED (37). A multicenter prospective study conducted in different countries showed that ED was closely associated with severe skin, muscle, or kidney involvement in SSc. The study further pointed out that severe ED is a common and often early-onset problem in male SSc patients (38). A case-control study comparing male SSc and rheumatoid arthritis (RA) patients showed that the incidence of ED was higher in the SSc group. The study included 43 SSc patients and 23 RA patients. 81% of the SSc group had ED, and there were no significant differences between the two groups in other confounding factors, such as nerve damage and diabetes, suggesting that SSc itself may be the main factor underlying ED (39).

SLE is characterized by an imbalance in the immune system and the erroneous attack on the body’s own tissues (40), which may contribute to multi-organ and systemic damage. Male patients with SLE often face challenges with the ED. A recent meta-analysis on SLE and male reproductive health indicated that the risk of ED in SLE patients is higher than that in the control group (OR = 7.44; 95% CI = 5.00–11.06, p < 0.001), suggesting that male SLE patients are more likely to experience sexual function problems (41). A multicenter cross-sectional study conducted in Latin America further supported this conclusion. Notably, the average age of patients in the SLE group was only 36.3 years old, suggesting that ED is not caused by aging but is an important complication of SLE itself (42). Another cross-sectional study also found that compared with the control group, the penile circumference, sperm concentration, and sperm motility of SLE patients were also reduced (43). Although connective tissue diseases are more common in women, the above studies still highlight the critical impact of connective tissue diseases on male ED, indicating a clear association between the two.

### 3.4 Other immune-mediated skin diseases

The characteristic of AD is polymorphic skin lesions and a tendency to exudation. Its most prominent clinical manifestations are severe itching and recurrent rashes (20). A case-control study explored the association between AD and ED. The study included a total of 3997 patients with ED as the case group, and 19985 subjects without a history of ED were matched as the control group. The results showed that men with a history of AD had a higher incidence of ED (OR = 1.60, 95% CI = 1.42-1.80, P < 0.001) (44).

BD is a small-vessel vasculitis characterized by recurrent oral ulcers, eye inflammation, genital ulcers, and skin lesions. Numerous studies indicate that BD may negatively impact male sexual function. A study involving 42 BD patients and 42 healthy controls showed that the International Index of Erectile Function (IIEF) score of the case group was lower than that of the control group (45). Another study on 72 sexually active male BD patients and 62 healthy controls also found the widespread presence of sexual dysfunction in the patient group (46). In addition, a study including 38 male BD patients recommended routine assessment of erectile function in such patients in clinical practice (47).

LS is a chronic inflammatory disease that commonly occurs in the genital area (48). A multicenter observational study aiming to evaluate sexual health recruited a total of 176 participants, including patients with LS, patients with pemphigus, and healthy controls (49). The results showed that patients with LS faced more challenges in sexual health than patients with pemphigus and the healthy population. Further research indicated that there was an association between the severity of LS and poor quality of life (QoL) in sexual aspects.

## 4 Potential mechanisms and multi-factor interactions between immune-mediated skin diseases and ED

**Figure 1:**
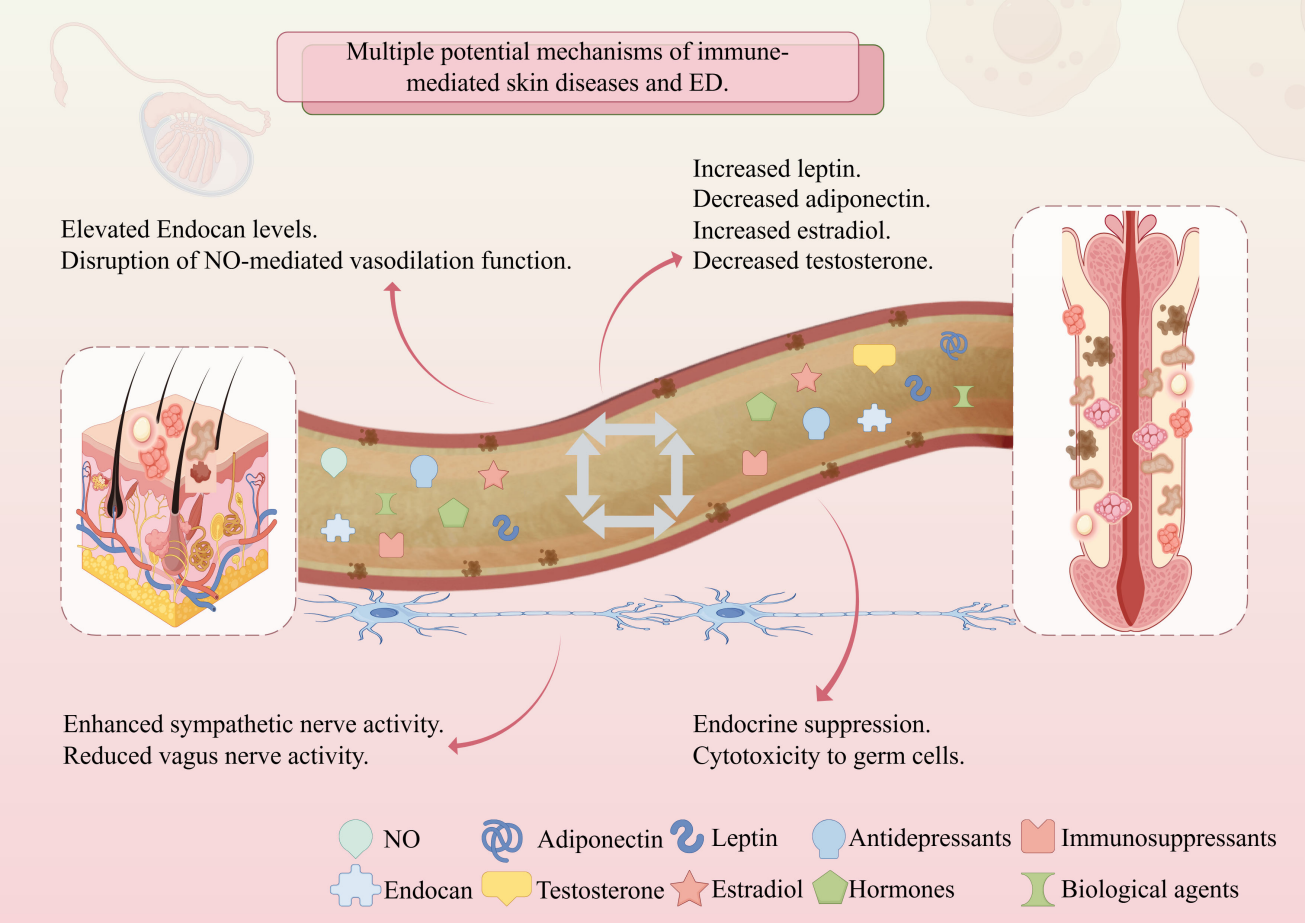
The impact of immune-mediated skin diseases on ED may occur through the following four main interrelated mechanisms. Chronic inflammation can damage the vascular endothelium and disrupt NO-mediated penile vasodilation. Metabolic abnormalities, such as adipokine imbalance and sex hormone disorders, may exacerbate inflammation, and vice versa. Neuropsychological factors affect erectile physiology through stress, dysregulation of the autonomic nervous system, and interactions within the HPA axis. Some therapeutic drugs may directly or indirectly interfere with sexual function, while effective control of inflammation can also bring about improvements. These mechanisms together form a multi-pathway interactive network. ED, erectile dysfunction; NO, nitric oxide; HPA, hypothalamus-pituitary-adrenal.

Although multiple observational studies have reported an association between immune-mediated skin diseases and ED, and the incidence of ED is relatively high among patients with such diseases, the specific mechanism is complex and has not been fully elucidated. This section aims to explore the potential pathways through which immune-mediated skin diseases affect ED, mainly including factors such as inflammation and endothelial dysfunction, metabolic disorders, neuropsychological regulation, and drug effects (**Figure 1**). These factors do not exist in isolation but are interrelated and act together, thereby increasing the risk of ED in patients. Understanding these potential mechanisms is expected to provide new targets for intervention in prevention and treatment (**Table 2**).

**Table 2:** Overview of some mechanisms underlying the association between major immune-mediated skin diseases and ED.

Disease	Mechanism	Key molecules/Examples of clinical findings	References
Psoriasis	Inflammation and endothelial dysfunction, metabolic disorder, nervous system and psychology	(1) Inflammation/Endothelium: Elevated Endocan levels. (2) Metabolism/Hormones: Often accompanied by obesity and metabolic comorbidities; disordered sex hormone levels. (3) Nerves/Psychology: Demyelinating polyneuropathy occurs after treatment with TNF-αinhibitors; perceived impairment of sexual function due to the disease.	(21, 24, 42, 54)
HS	Metabolic disorder	(1) Metabolism/Hormone: Prevalence of hypermetabolic syndrome	(70)
Scleroderma	Inflammation and endothelial dysfunction	(1) Inflammation/Endothelium: Penile hemodynamic abnormalities	(55, 56)
SLE	Drug effects	(1) Drugs: Men treated with cyclophosphamide had lower sperm concentration and total sperm count.	(43)
AD	Metabolic disorder	(1) Metabolism/Hormone: Prevalence of hypermetabolic syndrome	(71)
BD	Nervous system and psychology	(1) Neural factors: BD may affect the autonomic nerve center by involving the brainstem.	(72)
LS	Inflammation and endothelial dysfunction	(1) Inflammation: Commonly observed in chronic inflammatory conditions affecting the genital region.	(48)

ED, erectile dysfunction; HS, hidradenitis suppurativa; SLE, systemic lupus erythematosus; AD, atopic dermatitis; BD, behçet’s disease; LS, lichen sclerosus; TNF-α, tumor necrosis factor-α.

### 4.1 Inflammation and endothelial dysfunction

Chronic systemic inflammation is a hallmark of many immune-mediated skin diseases and is considered the main driving factor of endothelial dysfunction (50, 51), while endothelial dysfunction underlies the pathophysiology of ED (52, 53). Clinical studies have established a link between these immune-mediated skin diseases and markers of endothelial damage. For example, Tanasov et al. reported an association between these diseases and vascular endothelial dysfunction, providing clinical evidence for endothelium-dependent ED under inflammatory conditions (4). In patients with psoriasis complicated by ED, although there is currently a lack of direct quantitative data support, some studies have observed an elevated level of Endocan (54). Endocan is an important biomarker of endothelial dysfunction and inflammatory response (54), suggesting that inflammation may link skin inflammation, vascular endothelial damage, and the occurrence and development of ED at the molecular level by upregulating Endocan expression. Nevertheless, Endocan cannot currently be used as a biomarker for diagnosis or prognosis, and further research is needed to verify its value.

In addition to molecular markers, direct clinical assessment of vascular function also supports this association. Disease-specific evidence shows that patients with SSc complicated by ED often exhibit abnormal penile hemodynamics, including decreased peak systolic velocity (PSV), weakened flow-mediated dilation (FMD), and a decline in the cavernous artery Doppler index (55, 56), indicating that ED can be one of the manifestations of systemic vascular diseases. Moreover, theoretically, atherosclerosis triggered by chronic inflammation is another possible pathway linking immune-mediated skin diseases and ED. Although direct evidence for this in patients with skin diseases is limited, given that endothelial injury is the initiating step of atherosclerosis, this pathway is biologically plausible (57–59). Damage to endothelial function is considered to disrupt the nitric oxide (NO)-mediated vasodilation function, which is crucial for penile erection (60). Although the detailed molecular cascades (such as specific phosphorylation events) are mainly derived from non-skin immunological disease studies (61–63), the clinical evidence from serum biomarkers to imaging studies suggests that chronic inflammation-induced endothelial dysfunction is the core mechanism linking immune-mediated skin diseases and ED. The evidence for many connections in these pathways is indirect or inferential and still needs to be verified through future disease-specific studies.

### 4.2 Metabolic disorder

There is a close connection between immune-mediated skin diseases and metabolic disorders in the body. The two can act synergistically to increase the risk of ED. In addition to overall obesity, specific manifestations of metabolic syndrome, such as insulin resistance, dyslipidemia, and visceral fat accumulation, may also play a particular role in the development of ED. Insulin resistance can impair endothelium-dependent vasodilation (64). Dyslipidemia may affect erection by impairing the function of penile endothelial cells (65). Visceral adipose tissue, as an active endocrine organ, participates in the regulation of inflammation and metabolism by secreting adipokines. For example, elevated leptin has a pro-inflammatory effect. It may exacerbate endothelial dysfunction and oxidative stress (66), while adiponectin, which has anti-inflammatory and vascular-protective effects, often shows a downward trend in states of metabolic disorders (67). The disorder of these adipokine levels may constitute a key molecular link between metabolism-related inflammation and vascular endothelial damage.

This kind of inflammatory state and adipokine dysregulation may also interfere with the function of the hypothalamic-pituitary-gonadal axis and thus contribute to the pathogenesis of hypogonadism (68). In male patients with psoriasis, HS, and AD, a relatively high prevalence of obesity and related metabolic comorbidities can be observed (69–71), suggesting that metabolism-related mechanisms may play a certain role in the occurrence and development of their ED. For example, In male patients with psoriasis, sex hormone levels are often disordered, typically manifested by a decrease in total testosterone levels and an increase in estradiol, which may be an important reason for their susceptibility to ED (24). It is worth noting that for psoriasis patients with hypogonadism, exogenous testosterone replacement therapy can not only improve erectile function but also reduce the severity of psoriasis lesions, lower inflammatory indicators, and optimize metabolic parameters (69). This supports the notion that immune-mediated skin diseases may jointly promote the occurrence and development of ED by disrupting metabolic homeostasis, affecting sex hormone balance, and inflammatory responses.

### 4.3 Nervous system and psychology

The impact of immune-mediated skin diseases on ED may also be related to the complex and bidirectional “neuroimmune-psychological axis”. The chronic inflammation of these diseases and their visible external manifestations may constitute long-term stressors, drive neuroimmune remodeling, and simultaneously cause psychosocial burdens. These two factors influence each other and may ultimately jointly contribute to the development of ED.

At the neuroimmune level, pro-inflammatory cytokines may interact with cortisol rhythm disruption by interfering with the hypothalamus-pituitary-adrenal (HPA) axis, thereby maintaining or exacerbating the inflammatory state (73). In addition, the neurotoxic effects of these inflammatory cytokines are speculated to be related to autonomic nervous system dysfunction (74), which is usually manifested as increased sympathetic nerve activity and decreased vagus nerve activity. This autonomic nervous system imbalance may weaken the physiological inhibition of systemic inflammation and further affect the local vasomotor regulation of the penis (75–77). There are also specific neuro-pathway hypotheses for some diseases. For example, BD may affect the autonomic nerve center due to involvement of the brainstem (72). Individual literatures indicate that a minimal number of patients develop demyelinating polyneuropathy when treated with tumor necrosis factor-α (TNF-α) inhibitors for diseases such as psoriasis (78). Although the causal relationship has not been established and the incidence is extremely low, theoretically, it cannot be completely ruled out that if such neuropathy occurs, it may affect the nerve function in the pelvic region and potentially interfere with the erectile process. These observations provide some clues for the neuroimmune mechanism, but further verification is still needed.

At the psychosocial level, visible skin lesions, desquamation, and discomfort in the genital area (such as pain and itching) are often associated with phenomena such as patients’sense of stigma, reduced self-esteem, and social avoidance (23). Studies have shown that more than 90% of psoriasis patients feel less attractive and believe that the disease harms their sex life (21). Long-term physical and mental stress may increase the risk of depression and anxiety disorders, and these psychological states are not passive outcomes. They can exacerbate erectile difficulties through behavioral avoidance and physiological mechanisms. For example, anxiety may activate the sympathetic nerve, thereby inhibiting the erectile process (79). It is worth emphasizing that the neuroimmune and psychosocial pathways are not independent of each other. Chronic inflammation is related to symptoms such as apathy and anhedonia, and the state of depression and anxiety may, in turn, exacerbate the systemic inflammatory response through the HPA axis and the autonomic nervous system, thus forming a possible cyclic relationship (80).

### 4.4 Drug effects

Drugs used to control immune-mediated skin diseases may affect sexual function either through direct pharmacological actions (such as neuroendocrine interference and gonadal toxicity) or have a positive impact on it through indirect pathways (such as reducing systemic inflammation and improving endothelial function and psychological state). The strength of evidence related to different drugs varies, ranging from well-researched causal relationships (such as antidepressants) to case observations that are only hypothesis-generating (such as biological agents). The associations with relatively conclusive evidence are mainly concentrated in antidepressant drugs. Drugs acting on the serotoninergic system have been confirmed by multiple studies to be one of the causes of drug-induced ED (81, 82). For example, a Meta-analysis report showed that the incidence of sexual dysfunction associated with imipramine, citalopram, fluoxetine, paroxetine, sertraline, and venlafaxine ranged from 25.8% to 80.3% (83). Since patients often do not report actively due to shame, the actual incidence may be underestimated. This may be due to its direct interference with the nervous system.

Associations that are suggested by clinical studies but need further verification can be found in some immunosuppressants and hormones. A prospective study on male patients with psoriasis complicated by ED found that after 6 months of treatment with low-dose methotrexate (MTX, <15 mg/week), patients experienced a decrease in erectile function scores and a reduction in total testosterone levels (84). Its potential mechanism may stem from the potential cytotoxic effect of MTX on Leydig cells, which may reduce the number of Leydig cells and their luteinizing hormone (LH) receptor expression (85, 86). Regarding cyclophosphamide, research has mainly focused on its negative impact on male reproductive ability. A study on patients with SLE showed that the sperm concentration and total number of those receiving intravenous cyclophosphamide treatment were lower than those of non-users (43). Although there are few studies on its direct effect on erectile function, this suggests that clinicians should fully inform patients about the relevant risks when using it in men of reproductive age. Long-term and high-dose use of glucocorticoids may interfere with the function of the HPA axis, inhibit the release of gonadotropins, and then reduce testosterone levels, which may be related to the occurrence of ED (87, 88). These drugs may affect sexual function through direct endocrine inhibition and germ cell toxicity. However, effectively controlling immune-mediated skin disease activity may also confer indirect benefits for sexual function.

Currently, observations only at the level of case reports mainly involve some biological agents. There are very few case reports describing the occurrence of ED after using secukinumab, and the symptoms were relieved after switching to another interleukin-17 inhibitor (ixekizumab) (89). Such reports only indicate that there may be idiosyncratic reactions in extremely individual patients, but are far from establishing a general causal relationship for this type of drug. On the contrary, most clinical studies show that biological agents (such as adalimumab and dupilumab) can improve patients’ erectile function by effectively controlling skin and systemic inflammation (20, 90). In conclusion, when evaluating the impact of therapeutic drugs on ED, a differentiated perspective is needed. It is necessary to consider both the possible direct pharmacological side effects and the indirect benefits of controlling the primary disease, to carefully balance the overall risk of undertreatment and the potential adverse reactions of drugs in clinical decision-making.

## 5 Assessment tools and clinical screening

It is recommended to establish an active screening pathway for ED in male patients diagnosed with immune-mediated skin diseases. Since ED is a common comorbidity in this group and patients often avoid reporting it actively due to a sense of shame, all male patients with genital involvement, high disease activity, or subjective reports of decreased QoL should be included in the systematic assessment. Comprehensive screening should be carried out by combining self-assessment questionnaires and objective physiological examinations.

Patient self-assessment questionnaires have become essential tools for clinical screening and evaluation due to their non-invasive nature, high efficiency, and good reproducibility. Among them, the IIEF is currently a widely used assessment scale for evaluating ED. In multiple studies on psoriasis, SSc, BD, and HS (91–94), the IIEF, as a core assessment indicator, effectively revealed the high prevalence of ED in these patient populations. The scores of this scale can standardize the grading of the severity of ED, with higher scores indicating better erectile function (38). The Dermatology Life Quality Index (DLQI), a commonly used tool for assessing the QoL in dermatology, can serve as an efficient and direct initial screening method (21). The Skin-Related Sexual Life Questionnaire (SRSLQ), as a relatively new assessment tool, is also used to evaluate the specific sexual dysfunction and related psychological burdens of dermatology patients (95). Psychological assessment is also crucial, as depression and anxiety are common driving factors for both ED and skin diseases. The Beck Depression Inventory (BDI) is commonly used to assess psychological states. Studies have found that the BDI score is negatively correlated with the IIEF score (22), highlighting the value of psychological assessment in comprehensive etiological judgment.

If the questionnaire screening indicates the presence of ED, or when it is necessary to clarify the etiology, it is recommended to refer the patient to a urology specialist for further objective examinations. Penile Doppler ultrasonography (PDU) is an important standard for evaluating vascular ED, which can detect parameters such as PSV, end-diastolic velocity (EDV), and resistance index (RI) of the penile artery. In addition, there is a correlation between PDU indicators and IIEF scores as well as endothelial function markers (55, 56), providing an objective basis for the pathophysiological mechanism of ED. Hormone level testing is also helpful for screening endocrine etiologies. It is recommended to include total testosterone, free testosterone, FSH, LH, and prolactin. Other specialist examinations, such as the nocturnal penile tumescence test and neurophysiological evaluation, can be used at the discretion of the specialist doctor, according to the individual situation of the patient (72).

## 6 Treatment and management strategies

**Figure 2:**
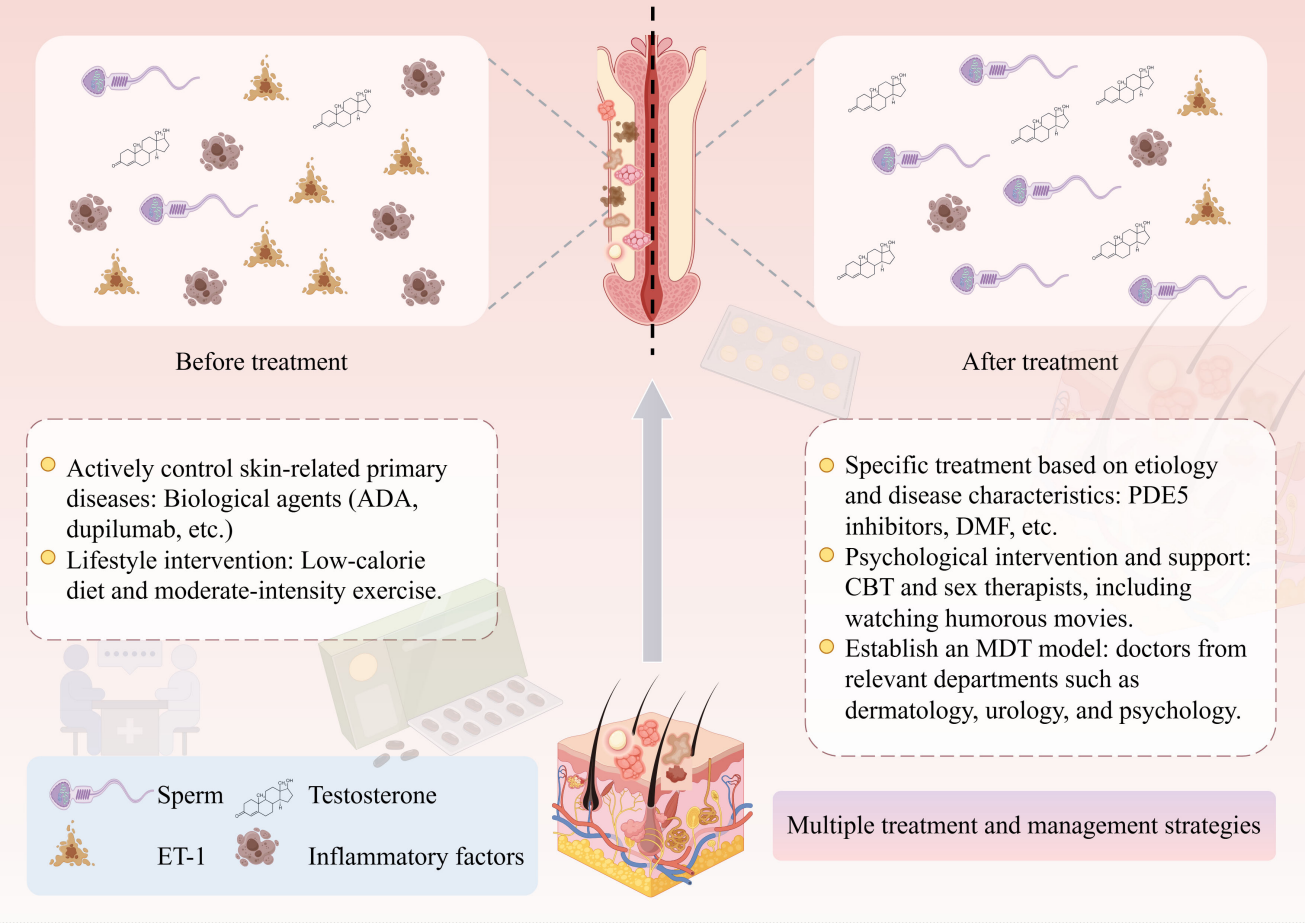
Management strategies for patients with ED. Actively controlling the primary disease is the cornerstone of improving ED, and individualized treatment plans need to be formulated according to the specific conditions of patients. Meanwhile, attention should also be paid to lifestyle intervention and psychological counseling. Ultimately, a comprehensive intervention system centered on patients, combining multiple modes and involving various disciplines, should be established. ED, erectile dysfunction; ADA, adalimumab; PDE5, phosphodiesterase 5; DMF, dimethyl fumarate; CBT, cognitive-behavioral therapy; MDT, multidisciplinary team; ET-1, endothelin-1.

As shown by the epidemiological evidence mentioned above, the prevalence and risk of ED are increased (OR range: 1.60-7.44) in male patients with various immune-mediated skin diseases (27, 41, 44). Therefore, a patient-centered, multi-modal, and multidisciplinary integrated strategy should be adopted for the management of ED in such patients. It not only emphasizes the active control of the primary immune-mediated skin diseases but also requires specific treatment for ED symptoms. Concurrent attention should be given to psychological support and social factors, alongside recommending scientifically grounded lifestyle modifications (**Figure 2**).

Given that chronic systemic inflammation is currently the core hypothesis explaining the association between immune-mediated skin diseases and ED, and the disease activity is related to the severity of ED, actively controlling primary cutaneous immune diseases is the primary measure to improve ED. As previously mentioned, in terms of treatment options, careful consideration should be given in combination with the effects of different drugs. Lifestyle interventions also have a positive effect. A randomized controlled trial showed that a 12-week low-calorie diet combined with moderate-intensity exercise can simultaneously improve the severity of psoriasis, ED, and metabolic syndrome (18).

The specific treatment of ED should be individualized according to the etiology and disease characteristics. Phosphodiesterase 5 (PDE5) inhibitors can promote smooth muscle relaxation and penile erection (96, 97). They have become the commonly used drugs for the treatment of most ED patients, but their efficacy varies with the disease. Studies have shown that for ED associated with SSc, the routine on-demand use of PDE5 has little effect (98). When switching to once-daily treatment with 10 mg of tadalafil for 12 consecutive weeks, it can not only improve the erectile function and IIEF scores of SSc patients, but also increase the PSV and reduce the plasma endothelin-1 (ET-1) level, which may have a potential effect on delaying the progression of penile fibrosis (99). Additionally, the exploration of new treatment methods is continually advancing. Animal experiments have shown that dimethyl fumarate (DMF) can improve diabetic ED in rats by alleviating oxidative stress and endothelial dysfunction (100), providing a new direction for future treatment. If the efficacy of drugs is insufficient, extracorporeal shock wave therapy (ESWT) can be combined (101). For patients with severe organic ED, penile prosthesis implantation can also be considered (102).

Given that the incidence rates of depression, anxiety, and inferiority complex among patients with immune-mediated skin diseases are much higher than those in the general population (23), psychological intervention and support are indispensable components. Patients with skin immune disorders often experience psychological issues such as body image concerns, depression, and anxiety, which can exacerbate ED. Healthcare providers should foster an open, non-judgmental communication environment in clinical settings, proactively inquire about patients’ sexual health, and clarify that ED is a common comorbidity of such conditions rather than a personal failure, thereby alleviating psychological burdens. Regarding intervention strategies, cognitive behavioral therapy and sex therapy are particularly crucial. Even simple interventions can improve ED symptoms in the short term and regulate related hormone levels, such as watching humorous movies (103), demonstrating the broad clinical potential of psychological interventions.

Establishing a multidisciplinary team (MDT) model is an essential guarantee for achieving optimized management. An ideal team should include dermatologists, urologists, and psychologists. Dermatologists are responsible for initial screening, assessment, and control of the primary disease; urologists focus on the specialized diagnosis and specific treatment of ED; psychologists handle emotions and related issues. Through this collaborative model, comprehensive and continuous care can be provided for patients, and their QoL and sexual health outcomes can be improved to the greatest extent.

## 7 Conclusion and outlook

By sorting out the associations between various immune-mediated skin diseases and ED, this paper breaks through the scope of previous single-disease reviews. Starting from an integrated perspective of cross-disease comparison, it systematically summarizes the common pathophysiological mechanisms and disease-specific factors related to the associations between these types of diseases and ED. Its pathogenesis is complex and multifaceted, encompassing multiple pathways, including systemic inflammation, microvascular lesions, endothelial dysfunction, hormonal abnormalities, neural factors, and psychosocial stress. These factors form an intertwined network that fully reflects the characteristics of the biopsychosocial medical model. In addition, it also reveals a clinical reality worthy of attention. Male patients with immune-mediated skin diseases have an increased burden of ED, and such sexual dysfunction is not an isolated problem. It is often closely related to a higher proportion of depression, anxiety, and a decline in QoL, causing multifaceted damage to patients’ health. Based on this, this review proposes a unified, operational multidisciplinary clinical management framework to promote the transition from single-disease management to holistic intervention for comorbidities. Active treatment of the primary disease should be emphasized, given that controlling the shared inflammatory drive is foundational. Biological agents can not only effectively control the activity of skin lesions but also improve ED symptoms, highlighting the therapeutic potential of targeting common pathways. The treatment of ED itself should be individualized according to the specific etiology, with careful consideration of the potential negative impact of socme immunosuppressants on sexual function, as well as the imperative to address frequent psychological comorbidities such as depression and anxiety. In addition, the optimization and adjustment of lifestyle also have an apparent improvement effect on ED.

To translate the current understanding into tangible clinical progress, future research should prioritize three interrelated directions. First, it is imperative to move beyond associative observations and elucidate the specific mechanistic pathways linking cutaneous immune dysregulation to penile vascular, neural, and endocrine dysfunction. This includes research on key inflammatory mediators and the validation of promising biomarkers, such as Endocan, for diagnosis and treatment monitoring. Second, large-scale, prospective clinical studies are urgently needed to establish causal relationships and evaluate interventions. Finally, research must focus on developing and optimizing personalized, multidisciplinary intervention models. This entails defining which patient subsets respond best to specific ED treatments within this unique population, and rigorously evaluating integrated care protocols that seamlessly combine dermatological management, urological treatment, psychological support, and lifestyle modification. Through a continuous process in clinical trials, from elucidating molecular mechanisms to validating targeted interventions, the field can move towards a proactive approach to precision medicine, effectively addressing the dual burden of immune-mediated skin diseases and ED, and ultimately restoring the QoL of affected individuals.
